# The effect of probiotics on the clinical status of adult patients with atopic dermatitis: a systematic review

**DOI:** 10.1186/s40001-022-00713-z

**Published:** 2022-06-15

**Authors:** Camila Stofella Sodré, Mayra Stambovsky Vieira, Juliany Lima Estefan, Cristiane Moraes, Fernanda Sampaio Cavalcante, Kátia Regina Netto dos Santos, Dennis de Carvalho Ferreira

**Affiliations:** 1 Faculty of Dentistry, Centro Universitário Universus Veritas, Rio de Janeiro, Brazil; 2 Faculty of Dentistry, Universidade Veiga de Almeida, Rio de Janeiro, Brazil; 3 Department of Clinical Medicine, Universidade Federal do Rio de Janeiro- UFRJ, Rio de Janeiro, Brazil; 4 Faculty of Dentistry, Universidade Estácio de Sá, Rio de Janeiro, Brazil; 5 Universidade Federal do Rio de Janeiro (UFRJ) - Campus Macaé, Rio de Janeiro, Brazil; 6 Microbiologia Paulo de Góes Institute, Universidade Federal do Rio de Janeiro - UFRJ, Rio de Janeiro, Brazil; 7 Faculty of Dentistry, Universidade Veiga de Almeida, Rio de Janeiro, Brazil

**Keywords:** Atopic dermatitis, Prebiotics, Probiotics, Synbiotics

## Abstract

**Objectives:**

To describe, through a literature review, the results and benefits of oral and topical probiotics for adult patients with atopic dermatitis.

**Design:**

A systematic review of articles published over a 13-year period was conducted to answer the following questions: (1) what information is given in the scientific literature concerning the use of probiotics in adult patients with atopic dermatitis? (2) Was there an improvement in the clinical status of the patients? (3) Was there a change in the microbial profile in patients after using such approaches? (4) Among the probiotics used, which was the most used in adult AD patients? (5) What was the average time of these interventions? (6) What were the outcomes?

**Results:**

Seven studies with different sample sizes, ranging from 16 to 109 patients, were included in this review. These studies were all clinical trials (7/7), and probiotics (7/7) was the model of intervention chosen. Probiotics showed a potential to relieve the symptoms of the study groups with a reduction of pruritus and SCORAD when compared to the placebo groups. However, their effectiveness varied according to the strain, period, and form of administration.

**Conclusions:**

Many studies have demonstrated that probiotics improve the symptoms of atopic dermatitis and even its prevention. However, there is still much controversy and divergence concerning the real benefits. Despite this, probiotics have demonstrated a fair ability in improving AD adult patients’ symptoms in terms of decreasing pruritus and severity related to SCORAD.

## Introduction

Atopic dermatitis (AD) [1] is characterized as one of the most common chronic cutaneous inflammatory disorders today and its prevalence has increased worldwide. It affects 10–20% of children and 1–3% of adults [2, 3]. A clinical diagnosis of AD is imperative and indispensable [4, 5] as there is still no laboratory marker that is specific for its diagnosis. Furthermore, its pathogenesis has not yet been fully explained as this involves the interaction of factors such as: defects in skin barrier function, immunological changes, psychological aspects (like stress) and environmental aspects, in addition to the participation of infectious agents [5].

The clinical approaches to individuals with AD are to avoid triggering factors, the use of topical calcineurin inhibitors and topical corticosteroids associated with moisturizers due to transepidermal fluid loss, to control itching with the use of antihistamines, and in refractory cases, immunosuppressants may be prescribed [6]. These medications, despite providing relief and some resolution of AD symptoms, are not a definitive treatment, and consequently, new therapies must be sought and investigated [6].

Some AD patients have food allergies due to the permeability of the small intestine. These allergies are commonly related to cow's milk, eggs and peanuts [6]. Such allergies strongly influence the degree of disease evolution and affect 35–40% of school-aged patients with AD; moreover, this is four times more prevalent in children under 5 years old [6, 7].

To understand these allergies in AD patients, it is necessary to know the microbial profile of AD. In healthy individuals, *Staphylococcus epidermidis* is predominant and, together with *Staphylococcus cohnii,* it could provide protection for the development of atopic dermatitis [8]. However, individuals with AD have a greater colonization of *Staphylococcus aureus.* This causes an increase in permeability of the skin barrier in individuals with active eczema, and an instability of the skin barrier, which lead to a chain reaction, and consequently an inflammatory process [8, 9], through the participation of inflammatory cells and immune responses with late (Th1) and immediate (Th2) phases [10–12].

The intestinal microbiota in healthy and AD individuals are also different. The intestinal flora of healthy individuals contains species of the *Lactobacillus* (phylum Firmicutes) and *Bifidobacterium* (phylum Actinobacteria) genera, while *Clostridium* (phylum Firmicutes) or *Staphylococcus* (phylum Firmicutes) are associated with atopic diseases. These latter two microorganisms are also more commonly found in children born by cesarean delivery [13, 14].

Due to these differences in intestinal microbiota, various studies have proposed the use of prebiotics, probiotics and/or synbiotics as therapies in the treatment of AD patients [15–18]. Probiotics are microorganisms that bring health benefits to the host if administered alive in certain amounts [19]. The organisms that are most used as probiotics are bacteria of the genera *Lactobacillus* and *Bifidobacterium*, and yeasts of the species *Saccharomyces boulardii* [20]. Most of these microorganisms are naturally part of the human enteric microflora, and, therefore, can survive gastric digestion. The main criteria for the choice of these probiotics are the fact that they can reach the intestines still alive and adhere to the walls [20].

Prebiotics are nutrients that promote the growth and/or activity of certain species of bacteria in the intestinal microbiota, and thus benefit the host [20]. Therefore, foods with prebiotics should have certain properties, such as specificity in relation to the bacteria to be targeted in the colon, the ability to increase the quality of the microflora benefiting the host and not be susceptible to absorption and hydrolysis in the upper digestive tract [20].

Synbiotics have both probiotic and prebiotic properties. This appropriate combination of both components makes them have a superior effect than probiotics or prebiotics alone [21, 22].

These alternative treatment therapies have demonstrated effectivity in treating a variety of conditions, including diarrhea, AD, and other chronic inflammatory conditions. The therapeutic potential is due in part to the effects of modulating the immune response, in addition to the competitive inhibition of microbiota components in the intestine and increased function of the epithelial barrier, which may help provide protection against the appearance of AD [15–18]. Also, some authors have suggested that early intervention during pregnancy could possibly be more effective than a late or postnatal intervention [23].

However, although several species have been considered effective in preventing or treating atopic dermatitis, the efficacy of probiotics in controlling AD is still uncertain [24]. Due to this controversy, the present study aims, through a systematic review of the literature, to describe the benefits and results generated by the use of probiotics, prebiotics and/or synbiotics in adult patients with AD. Furthermore, this review aims to clarify the possible changes that this alternative therapy promotes on the microbial profile of these patients, as well as discussing improvements in the clinical status of patients.

## Methodology

The methodology chosen for this systematic review was based on PRISMA (Preferred Reporting Items for Systematic Reviews and Meta-analyses) [25]. The following questions were asked to serve as the basis for this review: What information is given in the scientific literature concerning the use of probiotics in adult patients with atopic dermatitis? Was there an improvement in the clinical status of the patients? Was there a change in the microbial profile in patients after using such approaches? Among the probiotics used, which was the most used in adult AD patients? What was the average time of these interventions? What were the outcomes?

### Search strategy

**Fig.1:**
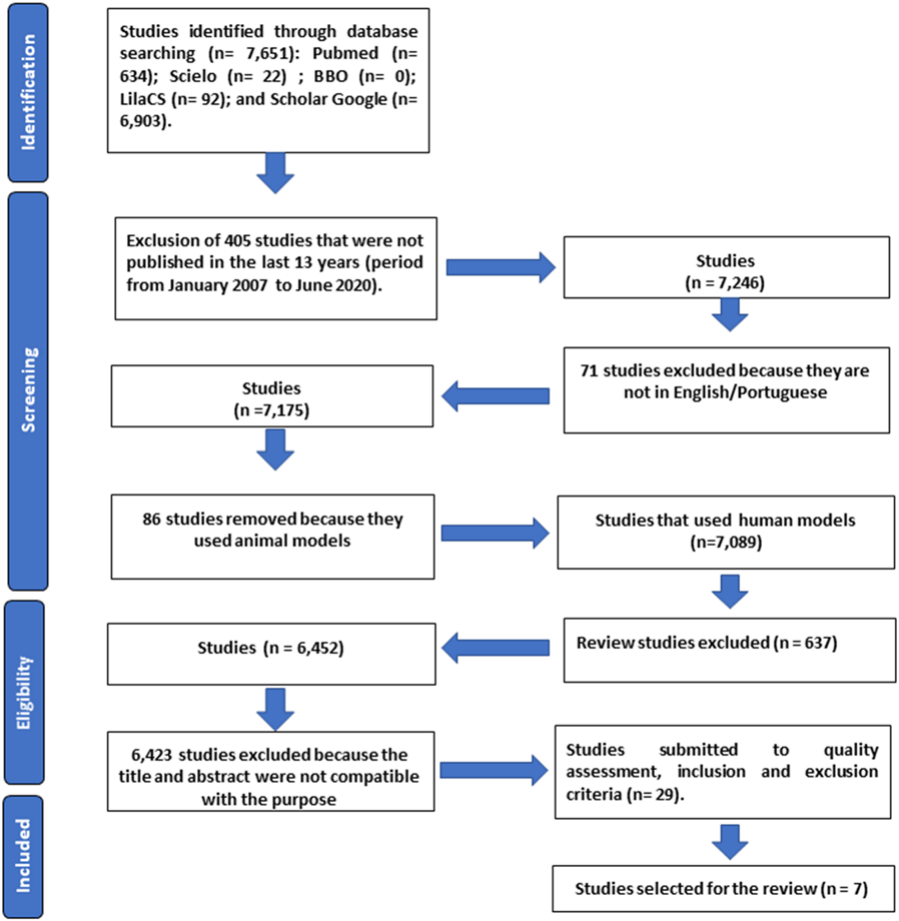
Flowchart of selection process. Database search was performed from January 2007 to June 2020

A flowchart (Fig. 1) of the bibliographic survey was based on two virtual health databases: PubMed (MEDLINE) and the Latin American and Caribbean Health Sciences Literature (LILACS); the Scientific Electronic Library Online (SciELO), and the Google Scholar search portal. The Boolean operators (AND, OR and NOT) were used and the intersection with the following keywords for the search "*atopic dermatitis*", "*probiotics*", "*prebiotics*", "*synbiotics*" were previously verified by the MEDLINE Mesh. The inclusion criteria selected original articles, which addressed the AD theme only for adults, and where the title and abstract agreed with the proposed theme; only publications in Portuguese and/or English were accepted; publications and/or *online* availability were taken from the period January 2007 to June 2020. The exclusion criteria were studies with animal models; theses; books; dissertations; patents and literature and/or systematic reviews.

### Population, intervention, control, outcomes (PICOs)

The population sample included patients aged 18 years or older with a diagnosis of AD who used probiotics. The Intervention was to evaluate the use of probiotics in adult patients with AD. The Control group consisted of systemically healthy adult patients without AD. The Outcomes reported were: (1) whether there was an improvement in SCORAD after treatment with probiotics in adult patients with AD; (2) if there was a change in the microbial profile after the use of probiotics in adult patients with AD; (3) the average time of interventions using probiotics.

### Study selection and quality assessment

Two independent reviewers selected the studies by crosschecking the keywords (C.S.S and M.S.V). Titles and abstracts were independently evaluated by the two reviewers (C.S.S and M.S.V), and duplicate studies, patents and articles that used animal models were independently excluded. When there was any disagreement between reviewers, a third reviewer determined the inclusion or exclusion of the study (J.L.E.).

The data of the included articles were summarized, and the methodology of the articles was analyzed using quality assessment (Table 1), based on the criteria established by PRISMA. Thus, depending on their score, studies showed high (8–10 points—level A), medium (5–7 points—level B) or low (0–4 points—level C) methodological quality. In this systematic review, only studies classified as level A and B were included. The risk of bias was also independently assessed using appropriate tools following the study design.

**Table 1:** Quality assessment of the selected studies for systematic review

Item	Quality assessment	Yes	No
1	Representative sample of the population	1	0
2	Definition of study type	1	0
3	Definition of inclusion and exclusion criteria for the target population	1	0
4	Presence of control group	1	0
5	Description of the period of probiotics and/or prebiotics and/or synbiotic administration	1	0
6	Description of the route of probiotics and/or prebiotics and/or synbiotic administration	1	0
7	Identification the SCORAD before and after probiotics and/or prebiotics and/or synbiotic treatment	1	0
8	Statistical analysis	1	0
9	Discussion of study limitations	1	0
10	Presence of ethics committee	1	0

The relevant findings of each selected study such as: first author, country and year of publication, database used, sample number, type of study, SCORAD, treatment used (probiotics) and route of administration were extracted from the original studies to build Table 2.

**Table 2:** Characteristics of the studies included in the systematic review

Author, country and year	Goals	Database/Keywords	Sample size	Age of patients	Inclusion of a restrictive diet	Type of study	Clinical results after using probiotics	Severity of the clinical SCORAD presented	Probiotics	Form of administration	Quality assessment
Roessler et al., Alemanha 2007 [31]	To investigate the influence of triple-lineage probiotic ingestion on clinical, immunological, and fecal parameters of AD patients and healthy adults	Pubmed/Atopic dermatitis; probiotics	Total sample (*n* = 30): 15 healthy adults 15 Patients with moderate AD	24 ± 3	No	Clinical trial	Presence of *L*. *paracasei* and *B. lactis* in the feces of patients after probiotic supplementation	Study showed improvement in SCORAD in patients with probiotic intervention in its 8 weeks of administration. There was no statistical difference between groups	*S. thermophilus* enriched with probiotic cultures *L. paracasei* Lpc-37, *L. acidophilus* 74–2 and *B. lactis* DGCC 420 for 8 weeks	Oral: daily Yogurt drink	A
Kaur et al., Estonia 2008 [33]	Investigation and regular use of emollients combined with ingestion of probiotics lessens the influence of environmental irritants and allergens that exacerbate inflammation and in AD patients	Google Scholar/Atopic dermatitis, probiotics	-Patients aged 20–42 years with mild to moderate AD (*n* = 16): 10 patients probiotic group 6 patients in placebo group	26 ± 6,4	No	Clinical trial	Reduction of oxidative stress and inflammation in the skin and blood after the use of probiotics	SCORAD reduction in the probiotic and placebo groups	*L. fermentum* ME-3 for 12 weeks	Oral: daily fermented goat milk with probiotic	A
Drago et al., Itália 2011 [32]	Evaluate the clinical and immunological effects of the ingestion of the probiotic *Lactobacillus salivarius* LS01 in the treatment of adult patients with severe and moderate atopic dermatitis	Pubmed/Atopic dermatitis, probiotics	Patients aged 18 to 46 years with moderate/severe AD (*n* = 38): 19 patients in the placebo group 19 patients in the treated group	30,46 ± 1,33	No	Clinical trial	Reduction of Th1 cytokines (IL-12 + IFNy) after 4 months (*p* = 0.03)	A significant reduction in SCORAD observed after the 4 months of probiotic use (*p* < 0.001)	*L. salivarius* LS01 in maltodextrin for 16 weeks, twice a day	Oral: twice a day. Sachets with the probiotic dissolved in water or another liquid according to the patient's preference	A
Moroi et al., Japão 2011 [28]	Investigate the clinical effect of a supplemental diet containing lactic milk *L. paracasei* K71 (LAB diet) in adult patients with atopic dermatitis (AD)	Pubmed/Atopic dermatitis, probiotics	Men and women with mild or moderate AD (*n* = 34)	20–65 a	No	Clinical trial	Reduction of pruritus in the 8 weeks of treatment (*p* = 0.059)	Did not use SCORAD, but Japanese dermatological association score. The skin severity score was lower at week 8 (*p* < 0.05) and at week 12 (*p* < 0.01)	*L. paracasei* K71 for 12 weeks once a day	Oral: Daily, Probiotic powder dissolved in water, coffee, or tea	B
Roessler et al., Alemanha 2012 [29]	Evaluate whether a probiotic mixture can affect the microbiota and its genotoxic activity in healthy individuals and patients with AD	Pubmed/Atopic dermatitis, probiotics	Healthy and AD patients (*n* = 30): 15 control group patients (age: 24 ± 3) 15 patients with AD group (age: 23 ± 3)	23 ± 3	No	Clinical trial	Increased *lactobacilli* in the stool of patients	SCORAD was only evaluated as an inclusion criterion for patients in their study	Probiotic drink containing: S. thermophilus enriched with *L. paracasei* Lpc-37; *L. acidophilus* 74–2 and *B. animalis subsp. lactis* DGCC 420 for 8 weeks	Oral: a daily probiotic drink	A
Matsumoto et al., Japão 2014 [30]	To evaluate the effects of *B. lactis* LKM512 in adult AD patients	Pubmed/Atopic dermatitis, probiotics	- Men and women with moderate or severe AD (*n* = 44): 22 control group patients (mean age 34.1 years); 22 patients in the study group (mean age: 33.5 years)	33, 5 (8,6)	No	Clinical trial	Administration of probiotics relieved itching in patients	SCORAD index was only evaluated before treatment	*B. lactis* LKM512 once a day for 8 weeks	Oral: daily with capsules	A
Fang et al., China 2020 [41]	Determine the effects of probiotics on clinical symptoms, immune responses and gut microbiota in AD patients	Pubmed/Atopic dermatitis; probiotics	109 mild AD patients: Placebo group (*n* = 26) Oligose group (*n* = 11), *Bifdobacterium bifidum* CCFM16 group (*n* = 29) *Lactobacillus plantarum* CCFM8610 group (*n* = 43)	Group CCFM8610:49.19 (14.59) CCFM16 Group: 53.57 (13.86)	No	Clinical trial	Improvement of symptoms with the probiotic *L. plantarum* CCFM8610	There were no significant differences in baseline characteristics between the four groups (*p* > 0.05) After 8 weeks of intervention, the group CCFM 8610 significantly decreased the SCORAD index	Oligosaccharides used: *Bifdobacterium bifidum* CCFM16 and *Lactobacillus plantarum* group CCFM8610 once daily for 8 weeks	Oral	A

The present review and the established criteria were based on the study previously published by Hulshof et al. [26].

## Results

In the initial search, a total of 7651 articles were found: 634 articles from PubMed, 92 articles from LilaCs, 22 articles from Scielo, and 6903 articles from Google Scholar. After applying the above-mentioned inclusion and exclusion criteria, the *Quality Assessment* according to PRISMA was carried out, and articles at levels A and B were selected. Then any duplicate articles were excluded leaving 7 articles for the present review (Table 2).

In terms of design, all studies analyzed were clinical trials (7/7) with a divergent sample size ranging from 15 to 109 patients. The choice of intervention in all these studies was probiotics (7/7). Studies with the use of synbiotics and prebiotics were not identified within the criteria established. The usage of the probiotics ranged from 8 to 16 weeks, with a mean of 10.28 weeks (SD 3.15).

All selected studies used the oral route of administration; however, the presentation of the probiotics varied. As shown in Table 2, the different forms were powder, yogurt, sachets, capsules, probiotic drink, and fermented milk.

Most studies (6/7) used the SCORAD [27] index (Table 2) to classify the severity of AD. The only study that did not use SCORAD was that of Moroi et al. [28]. These authors verified the severity of the disease using the score established by the Japanese Dermatological Association. In their results, the authors found an improvement in the patient's clinical status in 8 weeks, the skin severity score was lower (*p* < 0.05), and there were further improvements in 12 weeks (*p* < 0.01) [28].

The study by Roessler et al. [29], who used the SCORAD index, observed that before starting the probiotic intervention, the mean clinical SCORAD of 15 patients with AD was 24.0 and when analyzed again after 8 weeks of probiotics showed a reduction in SCORAD to 15.5 (p = 0.081; 95% CI 17.0–23.6). The authors also observed an improvement in skin conditions after the 8-week probiotic intervention in 10/15 patients with AD [29]. Differently from the other selected studies, Matsumoto et al. [30] used the SCORAD index as an inclusion criterion for patients and not in the clinical evaluation after using the probiotic *Bifidobacterium animalis *subsp* lactis* (LKM512- *B. lactis*) [30]. Matsumoto et al. concluded that this probiotic was effective in reducing itching, and thus improving the quality-of-life score as well as improvements in the other AD symptoms [30]. The other studies in this review also confirmed a positive clinical evolution after treatment with probiotics, such as a reduction in SCORAD [31, 34, 35] and improvement in pruritus [30].

The Materials and Methods of the studies included numerous analyses. Most were blood analysis to evaluate Immunoglobulin E (IgE) in the serum and determine the Interleukins (IL-12, IL-4 and IL-5). In addition, the collection and analysis of feces was also carried out to analyze any changes in the microbiota after the administration of probiotics. This latter analysis was performed in five studies (5/7) [29–33] among which were: Roessler et al*.* [31] who investigated the fecal concentrations of *Lactobacillus paracasei, Lactobacillus acidophilus* and *B. lactis*, and found a significant increase in the concentration of *L. paracasei* and *B. lactis* during the period of probiotic administration. Matsumoto et al*.* [30] found that the *B. lactis* was significantly higher in the probiotic group compared to the placebo individuals [30]. Both these studies used real-time PCR to carry out this analysis [30, 31].

On the other hand, Drago et al. [32] carried out the investigation of bacterial species through conventional PCR and reported a statistically significant decrease of *staphylococci* in the stools of the group treated with the probiotic *Lactobacillus salivarius LS01* (*p* < 0.05). This result suggests that this probiotic treatment may have an important role in the modulation of Thl/Th2 cytokine profiles and could be considered as an important adjuvant therapy in the treatment of adult AD [32].

In 2012, Roessler et al. [29] also analyzed bacterial species from the feces of patients with AD by quantitative PCR, in which the concentrations of short-chain fatty acids were measured using gas chromatography. The results showed that a probiotic supplementation provided a significant increase in *lactobacilli*, while the number of *bifidobacteria* and *bacteroidetes* remained unchanged. Although the fecal concentrations of short-chain fatty acids were not affected, fecal pH was significantly reduced from 7.0 to 6.6 in the AD patients after probiotic consumption.

Based on the subjects’ characteristics, the study by Roessler et al. [29] evaluated IgE, macrophage-derived chemokine (MDC) and IL-16 in patients with AD and compared these results with healthy subjects. The authors verified that these data were significantly elevated in patients with AD compared with the healthy subjects. Regarding diet, the dietary fiber uptake of AD and healthy subjects remained stable during the study [29]. In the other hand, Drago et al. [32] study mentioned clinical and epidemiological data of patients enrolled in probiotic and placebo treatment group. Some patients of these groups presented: respiratory allergy (14/19 patients of the placebo group; 16/19 patients of the probiotic group); food allergy (9/19 patients of the placebo group; 3/19 patients of the probiotic group); and serum IgE (919.71 ± 369.08 of the placebo group; 579.14 ± 253.63 of the probiotic group). Moroi et al. [28] study indicated the skin severity scores in both groups: *Lactobacillus paracasei* K71 (LAB diet group) and placebo group in different locations (head and neck, anterior trunk, posterior trunk, upper and lower limbs), but did not mention any epidemiological data of these patients [28]. Kaur et al. [33] evaluated AD patients that suffered from AD in their early childhood. The authors reported some data of the patients like family history of atopy (2/16) and presence of concomitant asthma and AD (1/16) in a few patients. Roessler et al. [29], Matsumoto et al. [30] and Fang et al. [41] did not mention any clinical and epidemiological data of patients enrolled in their study [29, 30]. Other important aspects of the selected studies are described in Table 3.

**Table 3:** Severity index, number of patients and use of topical treatments of the studies included in the systematic review

Author, country and year	Number of patients	Baseline SCORAD	SCORAD after treatment	Type of the control group (healthy controls or patients with AD without probiotics)	Topical treatment allowed?	Number of patients that completed the study
Roessler et al., Alemanha 2007 [31]	15 healthy subjects 19 patients with AD	24.0 [95% confidence interval (CI), 20.6–26.7]	21.9 (95% CI, 19.3–24.4) (8 weeks)	Healthy controls	Yes. The topical glucocorticoid Advantans (Scheming, Berlin, Germany) (1 mg/g) was administrated in the case of need	14 patients with AD (4 dropped out) and 15 healthy controls
Kaur et al., Estonia 2008 [33]	16 patients	Probiotic group: 4.8 ± 3.9 • Placebo group: 4.8 ± 2.8	Probiotic group: 1.9 ± 1.8 (12 weeks) Placebo group: 2.3 ± 0.9 (12 weeks)	Patients with AD without probiotics (placebo group)	Yes. The patients were allowed to use different emollient creams for everyday use (Eucerin®, Baierstorf, Hamburg; Decubal®, Dumex-Alpharma; Linola® fett N and Wolff Basis crème, Dr. August Wolff GmbH & Co, Arzneimittel, Germany; Aqualan® Orion Pharma)	16 patients
Drago et al., Itália 2011 [32]	38 adult patients	Probiotic group: 27.57 ± 3.4 Placebo group: 24.28 ± 2.15	Probiotic group: 13.14 ± 0.27 (16 weeks) Placebo group: 20.14 ± 0.27 (16 weeks)	Patients with AD without probiotics (placebo group)	Yes. The patients were allowed to use different emollient creams	38 patients
Moroi et al., Japão 2011 [28]	34 patients	Did not use SCORAD, but Japanese dermatological association score Probiotic group: 3.71 ± 1.76 Placebo group: 4.47 ± 2.00	Probiotic group: 1.00 ± 1.21 (12 weeks) Placebo group: d)0.71 ± 1.99 (12 weeks)	Patients with AD without probiotics (placebo group)	Yes. The patients were allowed to use topical corticosteroid and tacrolimus	34 patients
Roessler et al., Alemanha 2012 [29]	34 patients	SCORAD was only evaluated as an inclusion criterion for patients in their study Patients had a SCORAD score of 5–30	Not evaluated	Healthy controls	Did not mention	30 patients
Matsumoto et al., Japão 2014 [30]	44 patients	Probiotic group: 7.5(0.9) Placebo group: 7.6(0.9)	Not evaluated	Patients with AD without probiotics (placebo group)	Yes. The patients were allowed to use their medications as usual	44 patients
Fang et al., China 2020 [41]	120 patients	Placebo group: 19.62 (10.24) Oligose group: 17.31 (11.93) *Bifdobacterium bifidum* CCFM16 group: 21.64 (16.13) *Lactobacillus plantarum* CCFM8610 group: 18.79 (16.06)	The CCFM 8610 group significantly decreased the SCORAD index (8 weeks)	Patients with AD without probiotics (placebo group)	Did not mention	109 patients

## Discussion

Several studies have pointed to probiotic therapy as an alternative for the treatment of AD. This is because probiotics can modify the intestinal microbiota early in pregnancy and influence the development of adaptive immunity. In addition to these factors, they have immunomodulatory effects, which allow their use to be aimed at patients with AD who have low microbial diversity in the intestine during the first months of life [34–36].

This review obtained few findings in the literature, since testing of probiotics is more common in children and infants than in adults [35, 37–39]. The use of probiotics in children is more studied than in adults since the digestive tract of children is colonized by anaerobic bacteria, which can prevent allergic disorders [40]. The effect of probiotics on the intestinal composition of children is related to immature immune system, which can be more difficult in AD adult-type with a matured immune system. The symptoms of AD in adults can be alleviated by cell components presented in probiotic bacteria [30].

Another systematic review carried out previously by Hulshof et al. [26] selected 14 articles to assess the effect of dietary interventions with pre- and/or probiotics and synbiotics for the treatment of AD in children diagnosed with AD from 2008 to 2017. This review differs in several respects when compared to the present study, especially for the different type of sample evaluated (evaluation of children), and for having included studies investigating the topical administration route of probiotics, synbiotic and prebiotics.

On the other hand, like the present study, most studies (12/14) selected by those authors used the SCORAD index to assess the severity of AD. In their results, 3 out of 5 studies that used synbiotic intervention reported a reduction in AD severity after the dietary intervention compared to the control diet. This result was like the treatments that used probiotics, where there was an improvement in AD severity in 3 out of 4 studies. Hulshof et al. [26] observed that the effective administration time for dietary intervention with prebiotics, synbiotics and probiotics remains uncertain, and may vary from 8 to 24 weeks. Consequently, this may indicate that there is no association between the duration of the dietary intervention and the outcome of clinical improvement in AD [26]. In the present review, the time of consumption of probiotics ranged from 8 to 16 weeks.

Most of the studies in this present review described effective and beneficial results with probiotic treatments [28, 31–33, 41]. Drago et al. [32], in 2011, demonstrated a significant improvement in SCORAD (*p* < 0.0001) in adult AD patients with *L. salivarius* LS01 taken orally twice daily. The probiotic was in sachets in powder form to be dissolved in water or another liquid according to the patient's preference, for 16 weeks. In their results, the authors observed a reduction in staphylococci in feces after the treatment with probiotics [32]. However, the study by Roessler et al. [31] after 8 weeks did not find any significant improvement of SCORAD (*P* = 0.081) in patients treated with a combination of probiotics (*L. paracasei* Lpc-37, *L. acidophilus* 74–2 and *B. lactis* DGCC 420 [*B. lactis* 420]). However, this supplementation as a probiotic drink significantly increased the total number of fecal lactobacilli in both groups and a high number of *L. paracasei* and *B. lactis* was found, demonstrating good colonization of these organisms in the intestinal microbiota [31]. This difference in results between the two studies may be related to the different probiotics used as well as the period and strain used [42, 43].

A relevant factor to be considered in AD patients is the balance in the intestinal microbial composition, as these individuals tend to dysbiosis, especially favoring pathogenic microorganisms. There is evidence that probiotic bacteria can mediate beneficial effects by modifying the intestinal microbiota and its metabolic activity. Therefore, a supplemented mixture of probiotics can affect the peripheral immunological parameters, and relieve skin symptoms in patients with AD [44, 45]. However, Nutten [44] reported that despite the literature showing satisfactory results, there are still divergences regarding the use of probiotics in patients with AD as there is no standardization regarding the species of probiotics used, their dosages, time of use and the use of combined therapies [45, 46]. Thus, further research is needed to clarify the effectiveness of prebiotics and/or probiotics in improving the clinical status, as well as lead to a reduction of SCORAD in adult AD patients.

Four of the selected articles [32, 33, 41, 47] reported an improvement in SCORAD with the use of probiotics. The study by Moroi et al. [28] did not use the SCORAD as a criterion; however, they observed a reduction in the score of the Japanese Dermatological Association after the 8th and 12th weeks of patients undergoing treatment with conventional topical corticosteroids and tacrolimus. These authors analyzed stable symptomatic patients who used *L. paracasei* K71 powder dissolved in 100 ml of water once a day in their diet. The authors described that, symptomatic changes were assessed primarily by skin severity scores and secondarily by itching and impaired quality of life (QL) scores, collected at baseline and during weeks 4, 8, and 12 after the start of the intervention. In contrast, Kaur et al. [33] used a different methodology from other trials, where the patients, who were using emollients, consumed goat milk fermented with the antioxidative probiotic *Lactobacillus fermentum*. In addition to biochemical blood tests, they performed skin biopsies (4 mm) from the patients' backs before and after the study. The authors reported an improvement in skin condition, a decrease in SCORAD, and a reduction in oxidative stress in the skin and blood according to the oxidative stress marker (OxS) in their patients [33].

The study by Matsumoto et al. [30] was the only one that found an improvement in pruritus of the patients treated with the probiotic *B. lactis* LKM512 [30].

Roessler et al. [39], as presented in this review, used a probiotic drink containing *Streptococcus thermophilus*, enriched with *L. paracasei* Lpc-37, *L. acidophilus* 74–2 and *B. lactis* DGCC 420 for 8 weeks for adult AD patients. However, they did not observe an improvement in SCORAD or in the condition of the patients, and only reported an increase in fecal lactobacilli and a reduction in fecal pH with the supplemented probiotics [39].

Fang et al. [41] analyzed the microbial diversity by sequencing the next generation of the V3 and V4 regions of the 16S gene with the Illumina platform using the stool from patients with AD and their control group after 8 weeks of treatment with the probiotic *Lactobacillus plantarum* CCFM8610. The result of the sequencing analysis of the samples detected 23 phyla, of which four represented more than 98%. These four were Firmicutes, Actinobacteria, Bacteroidetes and Proteobacteria, with a predominance of Bacteroidetes (84%) and Firmicutes (86%). After the 8-week intervention, the relative abundance of the four phyla showed no variation between groups. However, in terms of the genus, there were changes in the intestinal microbiota. Before the intervention, there was a predominance of *Bacteroides* and *Blautia* in all groups; after the intervention, *Bacteroides spp., Prevotella spp., Lachnospiraceae* and *Ruminococcaceae* were dominant. In addition to these changes observed in microbial diversity, the authors also found a reduction in the SCORAD index, an improvement in the quality of life of patients and an increase in the expression of IL-10 in patients with AD, after the administration of the probiotic. Also, this approach was effective in preventing worsening skin condition of atopic dermatitis patients [41].

The studies that have used prebiotics in vivo and in vitro, have demonstrated an increase in the expressions of IL-10, IFN-γ, IgA and TGF-β [48, 49]. Furthermore, prebiotics are understood to induce the production of lactic acid in *Bifidobacterium* and *Lactobacillus.* Lactic acid has immunomodulating effects, and its fermentation by such microbial genera produces short-chain fatty acids, such as acetate, butyrate, and propionate. These metabolites serve as an energy source for colonocytes and are putative candidates for leukocyte ligands [50].

In addition to probiotics and prebiotics alone, synbiotics are combinations of prebiotics and probiotics, in which there is stimulation of the latter by the former [51, 52]. Although none of the studies evaluated in this review used synbiotics in their therapeutic approach with adult AD patients, there are some studies in literature that address the use of synbiotics in children with AD, but present conflicting result [53–55].

In general, probiotics have numerous advantages, as they can be targeted to treat health conditions, control gastrointestinal diseases as well as help lose weight. Despite these advantages, the FDA (Food and Drug Administration) has not yet provided guidelines or recommendations for the use of probiotics. However, for AD, a specific protective effect of probiotics in primary prevention has been observed over a long follow-up period, especially in children and during pregnancy [56].

## Conclusions

Even though various clinical trials have been carried out with the most diversified methodologies and different probiotic strains, their results are still conflicting, especially in terms of clinical improvement in adult patients with atopic dermatitis. In general, our analysis showed that probiotics demonstrate a fair ability to relieve symptoms of atopic dermatitis in adult patients reducing pruritus and decreasing SCORAD index.

## Data Availability

Not applicable.

## References

[1] Chamlin SL, Frieden IJ, Williams ML (2004). Effects of atopic dermatitis on young American children and their families. Pediatrics.

[2] Mayba JN, Gooderham MJ (2017). Review of atopic dermatitis and topical therapies. J Cutan Med Surg.

[3] Kumar MK, Singh PK, Patel PK (2014). Clinico - immunological profile and their correlation with severity of atopic dermatitis in Eastern Indian children. J Nat Sc Biol Med.

[4] Chernyshov PV (2013). Dermatological quality of life instruments in children. G Ital Dermatol Venereol.

[5] Akdis CA, Akdis M, Bieber T (2006). European Academy of Allergology and Clinical Immunology/American Academy of Allergy Asthma and Immunology: diagnosis and treatment of atopic dermatitis in children and adults: European Academy of Allergology and Clinical Immunology/American Academy of Allergy, Asthma and Immunology/practall consensus report. J Allergy Clin Immunol.

[6] Sicherer SH, Sampson HÁ (1991). Food hypersensitivity and atopic dermatitis: pathophysiology, epidemiology, diagnosis, and management. J Allergy Clin Immunol.

[7] Hyunjin L, Kyunghee S, Ran K (2013). Nutrient intake and food restriction in children with atopic dermatitis. Clin Nutr Res.

[8] Wollina U (2017). Microbiome in atopic dermatitis. Clin Cosmet Investig Dermatol.

[9] Wollenberg A, Kraft S, Oppel T (2000). Atopic dermatitis: pathogenic mechanisms. Clin Exp Dermatol.

[10] Leung DY, Nicklas RA, Li JT (2004). Disease management of atopic dermatitis: an updated practice parameter. Ann Allergy Asthma Immunol.

[11] Romagnani S (2000). The role of lymphocytes in allergic diseases. J Allergy Clin Immunol.

[12] Soumelis V, Reche PA, Kanzler H (2002). Human epithelial cells trigger dendritic cell mediated allergy inflammation by producing TSLP. Nat Immunol.

[13] Van Nimwegen FA, Penders J, Stobberingh EE (2000). Mode and place of delivery, gastrointestinal microbiota, and their influence on asthma and atopy. J Allergy Clin Immunol.

[14] Gerasimov SV, Vasjuta VV, Myhovych OO (2010). Probiotic supplement reduces atopic dermatitis in preschool children: a randomized, double-blind, placebo-controlled, clinical trial. Am J Clin Dermatol.

[15] Ghadimi D, Fölster-Holst R, de Vrese M (2008). Effects of probiotic bacteria and their genomic DNA on TH1/TH2-cytokine production by peripheral blood mononuclear cells (PBMCs) of healthy and allergic subjects. Immunobiology.

[16] Rose MA, Stieglitz F, Köksal A (2010). Efficacy of probiotic Lactobacillus GG on allergic sensitization and asthma in infants at risk. Clin Exp Allergy.

[17] Oelschlaeger TA (2010). Mechanisms of probiotic actions - a review. Int J Med Microbiol.

[18] Chapman CM, Gibson GR, Rowland I (2011). Health benefits of probiotics: are mixtures more effective than single strains?. Eur J Nutr.

[19] Nermes M, Kantele JM, Atosuo TJ (2010). Interaction of orally administered Lactobacillus rhamnosus GG with skin and gut microbiota and humoral immunity in infants with atopic dermatitis. Clin Exp Allergy.

[20] Van der Aa LB, Heymans HSA, Van Aalderen WMC (2010). Probiotics and prebiotics in atopic dermatitis: review of the theoretical background and clinical evidence. Pediatr Allergy Immunol.

[21] Markowiak P, Śliżewska K (2017). Effects of probiotics, prebiotics, and synbiotics on human health. Nutrients.

[22] Dissanayake E, Tani Y, Nagai K (2019). Skin care and synbiotics for prevention of atopic dermatitis or food allergy in newborn infants: a 2 × 2 factorial, randomized, non-treatment controlled trial. Int Arch Allergy Immunol.

[23] Cabridain C, Aubert H, Kaeffer B (2019). Effectiveness of an antenatal maternal supplementation with prebiotics for preventing atopic dermatitis in high-risk children (the PREGRALL study): protocol for a randomized controlled trial. BMJ Open.

[24] Yang HJ, Min TK, Lee HW (2014). Efficacy of probiotic therapy on atopic dermatitis in children: a randomized, double-blind Placebo-controlled Trial. Allergy Asthma Immunol Res.

[25] Moher D, Liberati A, Tetzlaff J, The PRISMA Group (2009). Preferred reporting items for systematic reviews and meta-analyses: the PRISMA statement. PLoS Med.

[26] Hulshof L, van’t Land B, Sprikkelman AB (2017). Role of microbial modulation of atopic dermatitis in children. Nutrients.

[27] European Task Force on Atopic Dermatitis (1993). Severity scoring of atopic dermatitis; the SCORAD index. Dermatol.

[28] Moroi M, Uchi S, Nakamura K (2011). Beneficial effect of a diet containing heat-killed Lactobacillus paracasei K71 on adult type atopic dermatitis. J Dermatol.

[29] Roessler A, Forssten SD, Glei M (2012). The effect of probiotics on faecal microbiota and genotoxic activity of faecal water in patients with atopic dermatitis: a randomized, placebo-controlled study. Clin Nutr.

[30] Matsumoto M (2014). Antipruritic effects of the probiotic strain LKM512 in adults with atopic dermatitis. Ann Allergy Asthma Immunol.

[31] Roessler A, Friedrich U, Vogelsang H (2007). The immune system in healthy adults and patients with atopic dermatitis seems to be affected differently by a probiotic intervention. Clin Exp Allergy.

[32] Drago L, Iemolp E, Nicolai RL (2011). Effects of Lactobacillus salivarius (DSM 22775) treatment on adult atopic dermatitis: a randomized placebo-controlled study. Int J immunopathol pharmacol.

[33] Kaur S, Kullisaar T, Mikelsaar M (2008). Successful management of mild atopic dermatitis in adults with probiotics and emollients. Cent Eur J Med.

[34] Abrahamsson TR, Jakobsson HE, Andersson AF (2014). Low gut microbiota diversity in early infancy precedes asthma at school age. Clin Exp Allergy.

[35] Gore C (2011). Treatment and secondary prevention effects of the probiotics Lactobacillus paracasei or Bifidobacterium lactis on early infant eczema: randomized controlled trial with follow-up until age 3 years. Clin Exp Allergy.

[36] Smith B, Bodé S, Skov TH (2012). Investigation of the early intestinal microflora in premature infants with/without necrotizing enterocolitis using two different methods. Pediatr Res.

[37] Enomoto T (2014). Effects of bifidobacterial supplementation to pregnant women and infants in the prevention of allergy development in infants and on fecal microbiota. Allergol Int.

[38] Williams HC, Burney PG, Hay RJ (1994). The U K working party's diagnostic criteria for atopic dermatitis. I. derivation of a minimum set of discriminators for atopic dermatitis. Br J Dermatol.

[39] Han Y, Kim B, Ban J (2012). A randomized trial of Lactobacillus plantarum CJLP133 for the treatment of atopic dermatitis. Pediatr Allergy Immunol.

[40] Isolauri E (2012). Development of healthy gut microbiota early in life. J Pediatr Child Health.

[41] Fang Z, Lu W, Zhao J (2020). Probiotics modulate the gut microbiota composition and immune responses in patients with atopic dermatitis: a pilot study. Eur J Nutr.

[42] Björkstén B (2001). The epidemiology of food allergy. Curr Opin Allergy Clin Immunol.

[43] Pelucchi C, Chatenoud L, Turati F (2012). Probiotics supplementation during pregnancy or infancy for the prevention of atopic dermatitis. Epidemiol.

[44] Notay M (2017). Probiotics, prebiotics, and synbiotics for the treatment and prevention of adult dermatological diseases. Am J Clin Dermatol.

[45] Iemoli E (2012). Probiotics reduce gut microbial translocation and improve adult atopic dermatitis. J Clin Gastroenterol.

[46] Nutten S (2015). Atopic dermatitis: global epidemiology and risk factors. Ann Nutr Metab.

[47] Yamamoto K, Yokoyama K, Matsukawa T (2016). Efficacy of prolonged ingestion of *Lactobacillus acidophilus* L-92 in adult patients with atopic dermatitis. J Dairy Sci.

[48] Hoentjen F, Welling GW, Harmsen HJM, Tannock GW (2005). Reduction of colitis by prebiotics in HLA-B27 transgenic rats is associated with microflora changes and immunomodulation. Inflamm Bowel Dis.

[49] Roller M, Rechkemmer G, Bernhard WB (2004). Prebiotic inulin enriched with oligofructose in combination with the probiotics *Lactobacillus rhamnosus* and *Bifidobacterium lactis* modulates intestinal immune functions in rats. J Nutr.

[50] Le Poul E, Loison C, Struyf S (2003). Functional characterization of human receptors for short chain fatty acids and their role in polymorphonuclear cell activation. J Biol Chem.

[51] van der Aa LB, Heymans HS, van Aalderen WM (2010). Effect of a new synbiotic mixture on atopic dermatitis in infants: a randomized-controlled trial. Clin Exp Allergy.

[52] Schrezenmeir J, de Vrese M (2001). Probiotics, prebiotics, and synbiotics—approaching a definition. Am J Clin Nutr.

[53] Chang YS, Trivedi MK, Jha A (2016). Synbiotics for prevention and treatment of atopic dermatitis a meta-analysis of randomized clinical trials. JAMA Pediatr.

[54] Kukkonen K, Savilahti E, Haahtel T (2007). Probiotics and prebiotic galactooligosaccharides in the prevention of allergic diseases: a randomized, double-blind, placebo-controlled trial. J Allergy Clin Immunol.

[55] Roze JC, Barbarot S, Butel MJ (2012). An a-lactalbumin-enriched and symbiotic-supplemented v. a standard infant formula: a multicentre, double-blind, randomised trial. Br J Nutr.

[56] Cao L (2015). Long-term effect of early-life supplementation with probiotics on preventing atopic dermatitis: a meta-analysis. J Dermatolog Treat.

